# Efficacy of metformin targets on cardiometabolic health in the general population and non-diabetic individuals: a Mendelian randomization study

**DOI:** 10.1016/j.ebiom.2023.104803

**Published:** 2023-09-19

**Authors:** Jie Zheng, Min Xu, Qian Yang, Chunyan Hu, Venexia Walker, Jieli Lu, Jiqiu Wang, Ruixin Liu, Yu Xu, Tiange Wang, Zhiyun Zhao, Jinqiu Yuan, Stephen Burgess, Shiu Lun Au Yeung, Shan Luo, Emma L. Anderson, Michael V. Holmes, George Davey Smith, Guang Ning, Weiqing Wang, Tom R. Gaunt, Yufang Bi

**Affiliations:** a Department of Endocrine and Metabolic Diseases, Shanghai Institute of Endocrine and Metabolic Diseases, Ruijin Hospital, Shanghai Jiao Tong University School of Medicine, Shanghai, 200025, China; b Shanghai National Clinical Research Center for Metabolic Diseases, Key Laboratory for Endocrine and Metabolic Diseases of the National Health Commission of the PR China, Shanghai Key Laboratory for Endocrine Tumor, State Key Laboratory of Medical Genomics, Ruijin Hospital, Shanghai Jiao Tong University School of Medicine, Shanghai, 200025, China; c MRC Integrative Epidemiology Unit (IEU), Bristol Medical School, University of Bristol, Oakfield House, Oakfield Grove, Bristol, BS8 2BN, United Kingdom; d Clinical Research Center, The Seventh Affiliated Hospital, Sun Yat-sen University, Shenzhen, Guangdong, 518107, China; e Center for Digestive Disease, The Seventh Affiliated Hospital, Sun Yat-sen University, Shenzhen, Guangdong, 518107, China; f Guangzhou Women and Children Medical Center, Guangzhou, Guangdong, 510623, China; g Division of Epidemiology, The JC School of Public Health & Primary Care, The Chinese University of Hong Kong, Hong Kong, China; h MRC Biostatistics Unit, Cambridge Institute of Public Health, Cambridge, CB2 0SR, United Kingdom; i Cardiovascular Epidemiology Unit, Department of Public Health and Primary Care, University of Cambridge, Cambridge, United Kingdom; j School of Public Health, Li Ka Shing Faculty of Medicine, The University of Hong Kong, Hong Kong SAR, China; k Division of Psychiatry, Faculty of Brain Sciences, University College London, London, United Kingdom; l NIHR Biomedical Research Centre at the University Hospitals Bristol NHS Foundation Trust and the University of Bristol, United Kingdom

**Keywords:** Metformin targets, Cardiometabolic diseases, General population, Non-diabetic individuals, Mendelian randomization

## Abstract

**Background:**

Metformin shows beneficial effects on cardiometabolic health in diabetic individuals. However, the beneficial effects in the general population, especially in non-diabetic individuals are unclear. We aim to estimate the effects of perturbation of seven metformin targets on cardiometabolic health using Mendelian randomization (MR).

**Methods:**

Genetic variants close to metformin-targeted genes associated with expression of the corresponding genes and glycated haemoglobin (HbA_1c_) level were used to proxy therapeutic effects of seven metformin-related drug targets. Eight cardiometabolic phenotypes under metformin trials were selected as outcomes (average N = 466,947). MR estimates representing the weighted average effects of the seven effects of metformin targets on the eight outcomes were generated. One-sample MR was applied to estimate the averaged and target-specific effects in 338,425 non-diabetic individuals in UK Biobank.

**Findings:**

Genetically proxied averaged effects of five metformin targets, equivalent to a 0.62% reduction of HbA_1c_ level, was associated with 37.8% lower risk of coronary artery disease (CAD) (odds ratio [OR] = 0.62, 95% confidence interval [CI] = 0.46–0.84), lower levels of body mass index (BMI) (β = −0.22, 95% CI = −0.35 to −0.09), systolic blood pressure (SBP) (β = −0.19, 95% CI = −0.28 to −0.09) and diastolic blood pressure (DBP) levels (β = −0.29, 95% CI = −0.39 to −0.19). One-sample MR suggested that the seven metformin targets showed averaged and target-specific beneficial effects on BMI, SBP and DBP in non-diabetic individuals.

**Interpretation:**

This study showed that perturbation of seven metformin targets has beneficial effects on BMI and blood pressure in non-diabetic individuals. Clinical trials are needed to investigate whether similar effects can be achieved with metformin medications.

**Funding:**

Funding information is provided in the Acknowledgements.

**Research in context**

**Evidence before this study**

We searched PubMed, Embase and clinicaltrials.gov databases from inception up to July 11, 2022 using the search terms: “metformin”, “body mass index [BMI]”, “coronary artery disease [CAD]”, “systolic blood pressure [SBP]”, “diastolic blood pressure [DBP]” “blood lipids”, “Mendelian randomization” and “clinical trials”, without language restrictions. 117 MR and trial studies have investigated the role of metformin on cardiometabolic outcomes in individuals with diabetes, establishing metformin's effect on body mass index and heart disease, but studies examining metformin effects on blood pressure are under powered. Little has been done to estimate the generalizability of metformin effects on cardiometabolic outcomes—for instance, in the non-diabetic population.

**Added value of this study**

We confirmed the averaged beneficial effect of seven known metformin targets on reducing CAD risk, and reducing BMI, SBP and DBP levels. Mitochondrial complex I showed the strongest target-specific effects on CAD, BMI and DBP. The one-sample MR analysis in UK Biobank suggested that metformin targets also showed robust beneficial effects on cardiometabolic outcomes in non-diabetic individuals.

**Implications of all the available evidence**

Our study provides two pieces of evidence that may influence clinical decision making: (i) our findings support a beneficial effect of seven known metformin targets on cardiometabolic health in the general population and in non-diabetic individuals; (ii) Metformin targets showed protective effects on BMI, SBP and DBP control in non-diabetic individuals. Clinical trials are needed to investigate whether metformin medication (which may have additional targets than the seven studied here) can achieve similar reductions in cardiometabolic disease risk for individuals with high risk of developing diabetes, e.g. those with pre-diabetes.

## Introduction

Given the intersection and co-morbidity between type 2 diabetes (T2D) and cardiovascular disease, the efficacy and safety of anti-diabetic therapies on cardiometabolic health are of importance.^1^ Metformin is the most widely used first-line anti-diabetic therapy that is taken by over 150 million people each year,^2^ which shows good safety profiling on cardiovascular diseases. To date, clinical trials of metformin have provided evidence to support its beneficial effects on several cardiometabolic diseases in individuals with diabetes, including coronary death, major cardiovascular disease and body weight,^3^ but its effect on blood pressure was unclear.^4^ Until now, few studies have investigated the generalizability of metformin effects on cardiometabolic diseases in the general population, where non-diabetic individuals with normal levels of glycated haemoglobin (HbA_1c_) levels are the majority.^5^^,^^6^ A study that estimates the average and target-specific causal effects of known metformin targets, and establishes the HbA_1c_ stratified effects of metformin targets will provide timely evidence to support the understanding of the HbA_1c_ lowering effects of metformin targets on cardiometabolic health in the general population, especially in non-diabetic individuals.^7^

Mendelian randomization (MR) is an epidemiologic approach that uses germline genetic variants as instruments to estimate the causal effect of a modifiable exposure on an outcome.^8^ MR has previously been used to evaluate drug efficacy^9^ and inform potential safety concerns of approved drugs.^10^ The effects of two metformin targets, AMP-activated protein kinase (AMPK) and growth differentiation factor 15 (GDF15), on cardiovascular diseases have been studied.^11^^,^^12^ However, the general effect of metformin is influenced by multiple pharmacological targets, including, but not limited to AMPK,^13^ mitochondrial complex I (MC1),^14^ mitochondrial glycerol 3 (MG3),^15^ GDF15^16^ and glucagon-like peptide-1 (GLP1),^17^ fructose bisphosphatase-1 (FBP1)^18^ and adenylyl cyclase (ADCY1).^19^ Ideally, we would like to reliably estimate the effect of metformin on cardiometabolic health, to highlight potential clinical uses, other than for diabetes treatment/management, and to inform design of clinical trials. To do this reliably, we would firstly need to know all metformin targets, and secondly be able to instrument them with genetic variants. These metformin targets are currently known and can be instrumented genetically. There are likely other targets that are yet unknown and/or cannot be instrumented. Thus, the current best estimate of the effects of metformin is to examine target specific effects to understand whether particular metformin targets are more important for a certain cardiometabolic disease, and this may also inform target-specific drug development. Novel molecular phenotypes, such as gene/protein expression data,^20^ and new methods, such as genetic colocalization,^21^ have been proposed to select reliable genetic instruments for drug targets^12^ such as metformin targets.^22^

The aim of this study was to estimate the averaged and target-specific effects of seven metformin targets on eight cardiometabolic phenotypes in the general population using two-sample MR. This study also attempted to investigate the effect of metformin targets on cardiometabolic health in non-diabetic individuals using one-sample MR.

## Methods

### Study design

**Fig. 1:**
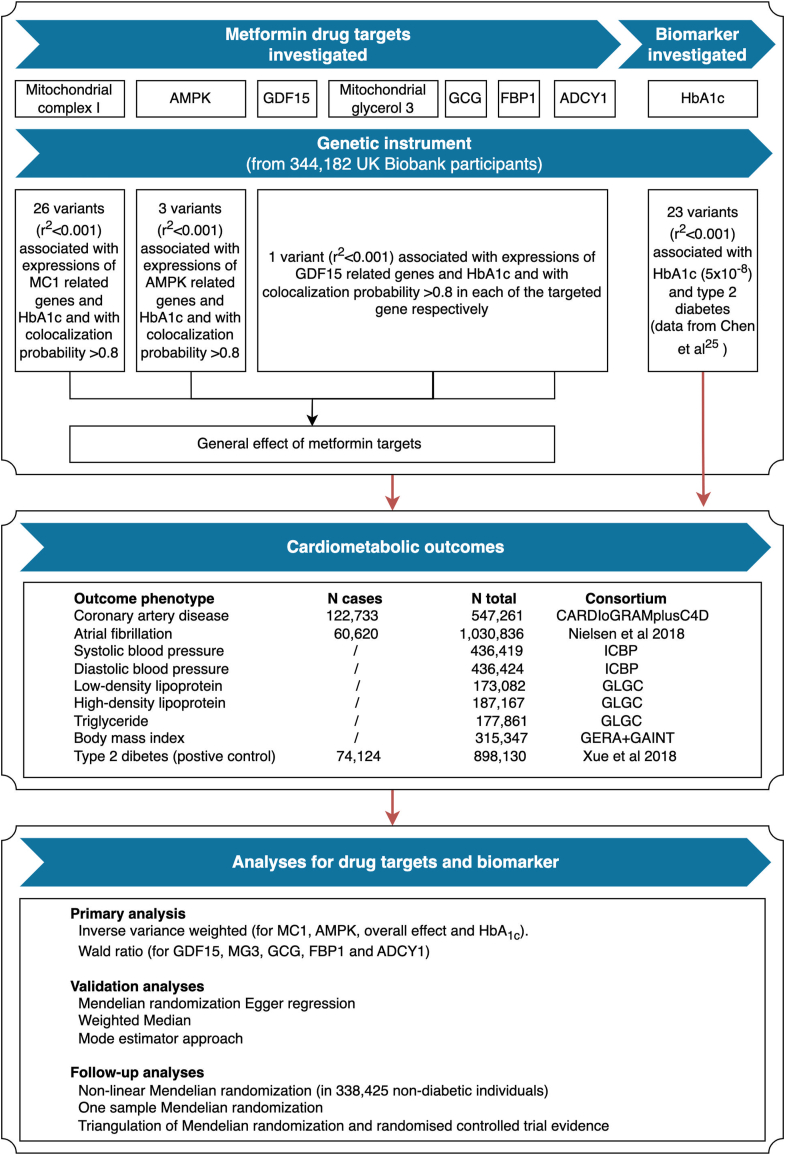
**Genetic instrument selection, data sources, and analysis strategy in a study of the lifelong effect of genetically proxied perturbation of metformin targets on cardiometabolic phenotypes**.

Fig. 1 presents a diagram of our genetic instrument selection (Supplementary Tables S1–S6), data sources, and the main and follow-up analyses. All studies providing data to this analysis had the relevant institutional review board approval from each country and all participants provided informed consent. Summary results were obtained from genome-wide association studies (GWAS) of HbA_1c_ (N = 344,182) and eight cardiometabolic outcomes (average N = 466,947; Supplementary Table S7).

Supplementary Fig. S1 illustrates the causal questions being interrogated in this study: (i) the average and target-specific effects of metformin targets on the eight cardiometabolic outcomes in the general population; (ii) the effects of circulating HbA_1c_ on these outcomes (as a benchmark analysis); (iii) the effects of metformin targets on these outcomes in non-diabetic individuals.

### Selection of genetic instruments for metformin targets and HbA_1c_

We generated genetic instruments to proxy the lifelong effect of seven metformin targets (AMPK, MC1, MG3, GDF15, GLP1/GCG, FBP1 and ADCY1) and of changes in HbA_1c_ levels. To select instruments for perturbation of metformin targets, we applied a conventional instrument selection process that has been used in previous drug target MR.^12^^,^^22^ In summary, this process selected genetic variants that:

(i) associated with both expression levels of corresponding genes (P < 0.01; N ≤ 31,684, data from GTEX,^20^ eQTLGen^23^ and Zheng et al.^24^) in the *cis*-acting regions (500 kb window from the centinal variant) and HbA_1c_ levels (P < 0.05; N = 344,182, data from UK Biobank);
(ii) showed evidence of genetic colocalization between the expression levels and HbA_1c_ levels within the corresponding genomic regions (colocalization probability >0.7);
(iii) passed a stringent linkage disequilibrium (LD; which is the pairwise squared correlation between nearby variants) r^2^ threshold of 0.001, which implies independent instruments to proxy perturbation of metformin targets.
(iv) have minor allele frequency over 1%.

A total of 26 variants proxying MC1, three variants proxying AMPK, one variant proxying GDF15, one variant proxying MG3, one variant proxying GCG/GLP1, one variant proxying FBP1 and one variant proxying ADCY1 were selected as instruments for the seven tested metformin targets respectively (Supplementary Table S6a; details in Supplementary Note 1). To instrument circulating HbA_1c_ levels, we selected 23 independent (r^2^ < 0.01) variants associated with both HbA_1c_ (data from MAGIC^25^) and type 2 diabetes (P < 5 × 10^−8^; data from Mahajan et al.^26^) (Supplementary Table S6b).

### Study outcomes

We selected cardiometabolic outcomes that are currently undergoing clinical trials for metformin use as outcomes for the MR analyses. We searched for the word ‘metformin’ in the CHEMBL^27^ and clinicaltrials.gov databases. This identified eight diseases/phenotypes with clinical trial records using metformin as treatment (coronary artery disease [CAD], systolic blood pressure [SBP], diastolic blood pressure [DBP], low-density-lipoprotein cholesterol [LDL-C], high-density-lipoprotein cholesterol [HDL-C], triglyceride, body mass index [BMI], atrial fibrillation; Supplementary Table S7). Type 2 diabetes was considered as a validation outcome. The genetic associations for these eight cardiometabolic outcomes and T2D were extracted from GWAS studies with an average of 466,947 samples per outcome, which were among the largest available studies for these outcomes to date.^28^ We noticed that the SBP and DBP GWAS included BMI as a covariate in the regression model.^29^ To avoid the issue of different covariates in the exposure and outcome,^30^ we used UK Biobank SBP and DBP GWAS (not adjusted for BMI in the model) as a validation analysis.

### Statistical analyses

Germline genetic variants used to proxy the perturbation of each of the metformin targets were matched to the eight cardiometabolic outcome datasets by harmonizing effects to the same effect allele and excluding palindromic variants with minor allele frequencies over 0.4. If an instrument was not available in the outcome dataset, a genetic variant in high LD (r^2^ > 0.8) with the instrument was selected as a proxy instrument. For the tested metformin targets, MR estimates were first generated per individual variant using the Wald ratio and standard errors were estimated using the delta method. A random-effects inverse-variance weighted meta-analysis was then used to combine variant-level Wald ratio estimates into a weighted-average effect estimate representing the overall HbA_1c_ lowering effect via metformin targets. All MR estimates (odds ratios [ORs] for analyses of binary outcomes and beta coefficients for analyses of continuous outcomes) were scaled to represent an SD unit of HbA_1c_ lowering. This reflects the equivalent of a 0.62% reduction in HbA_1c_.

In the main MR analysis, the average effects of the HbA_1c_ lowering effect of five metformin targets (proxied by all 34 metformin instruments together) on the eight cardiometabolic outcomes were estimated. For target-specific analysis, the specific effects of each of the metformin targets (i.e. seven targets as seven separate exposures) on the eight cardiometabolic outcomes were estimated. As a benchmark, the effects of circulating HbA_1c_ levels on the eight cardiometabolic outcomes were estimated to understand the influence of glucose on cardiometabolic outcomes. In this study, negative betas refer to a lower value of the outcome trait caused by genetically proxied lower HbA_1c_ levels, as this represents the potential effect of metformin. Likewise, for binary outcomes, an odds ratio below 1 refers to the protective effect of genetically proxied lower HbA_1c_ levels.

### One-sample MR and triangulation analyses

For MR estimates with evidence to support the HbA_1c_ lowering effect of metformin targets on a cardiometabolic outcome (Bonferroni-corrected threshold P < 0.006), we conducted extensive follow-up analyses of these outcomes in non-diabetic participants from UK Biobank (identified by excluding individuals with ICD-10 codes E10, E11, E12, E13, E14 and O24). These outcomes included BMI (UK Biobank ID: 21,001, unit kg/m^2^), SBP (UKBB ID: 4080; unit mmHg) and DBP (UKBB ID: 4079; unit mmHg). First, we conducted a one-sample MR to estimate the effects of perturbation of metformin targets on BMI, SBP and DBP in 338,425 non-diabetic participants. The genetic scores of the metformin targets are listed in Supplementary Table S6a (Supplementary Note 2). The effect of metformin targets on HbA_1c_ was estimated as a validation of the metformin instruments. The weighted average effect of metformin targets (34 instruments) as well as the target-specific effects (each target as an independent exposure) on BMI, SBP and DBP were estimated. CAD passed the linear MR threshold but was not considered as an outcome in these analyses due to the limited number of incident cases in the UK Biobank (N cases = 8891). Second, we triangulated genetic evidence from MR, pharmacoepidemiologic and trial evidence from the literature to validate the metformin effects on BMI,^31^ SBP and DBP^32^ (Supplementary Note 3).

In addition, since one-sample MR may overfit the data, we applied recently proposed two-sample MR approaches using single large-scale cohort,^33^ which may provide more accurate causal estimates. The 34 metformin drug target instruments were derived from separate dataset to avoid issue of winner's curse, with genetic associations information of HbA_1c_, BMI, SBP and DBP derived from the same 338,425 non-diabetic participants from UK Biobank. The inverse variance weighted, weighted median, weighted mode and Wald ratio two-sample MR methods were applied for this analysis.

### Validation of Mendelian randomization assumptions

In this study, we report findings according to the STROBE-MR (Strengthening the Reporting of Mendelian Randomization Studies) guidelines.^34^ MR has three key assumptions (Supplementary Fig. S2): (i) the germline genetic instruments used to proxy the drug targets are robustly associated with the exposure (“relevance”); (ii) the instruments are not associated with common causes (confounders) of the instrument-outcome relationship (“exchangeability”); (iii) the instruments are only associated with the outcome through the exposure under study (“exclusion restriction”). These MR assumptions were tested using a set of sensitivity analyses, although the “exchangeability” and “exclusion restriction” assumptions cannot be shown to hold directly.

The relevance assumption was validated by estimating the strength of the genetic predictors for each metformin target using the proportion of variance in each exposure explained by the predictor (R^2^) and F-statistics using the approximate approach. F-statistics above 10 are indicative of evidence against weak instrument bias. The exclusion restriction assumption was tested using a set of sensitivity analyses which relied on different assumptions. First, HbA_1c_ is a long-term blood glucose biomarker that also associated with red blood cell traits. Therefore, red blood cell traits may create a potential pleiotropic pathway linking metformin targets with cardiometabolic outcomes. To avoid this issue, we conducted a multivariable MR of metformin targets plus blood cell counts on cardiometabolic outcomes (Supplementary Table S6c). In contrast to univariable MR (which estimates a total causal effect), multivariable MR estimates the direct effect of an exposure of interest, by accounting for the indirect effect via secondary exposures. Second, we conducted a set of pleiotropy robust methods, including phenome-wide association study of instruments using PhenoScanner^35^ (Supplementary Table S6d), weighted median analysis, and simple and weighted mode estimator analyses. The heterogeneity between instruments was estimated using Cochran's Q test. A single variant MR comparison was carried out to examine whether MR estimates were driven by a single influential variant in drug target proxies. All these sensitivity methods were conducted using functions implemented in the TwoSampleMR package.^36^

For all analyses, the MR effect estimates were odds ratio (OR) with 95% confidence interval (CI) and relevant P values. A conservative Bonferroni-corrected threshold was used to account for multiple testing. In total, one exposure (averaged HbA_1c_ lowering effect of metformin targets) was tested against eight cardiometabolic outcomes (0.05/8 statistical tests; P value threshold = 0.006).

### Ethics

No additional ethical approval was required for the present study, since all analyses were based on publicly available summary statistics or individual level data from UK Biobank under application (17,295). The included GWAS studies all received informed consent from the study participants and have been approved by pertinent local ethical review boards.

### Role of funders

The funders were not involved in any activities of the current study, including study design, data collection, data analyses, interpretation, or writing of report.

## Results

### Strength of the genetic predictors of the perturbation of metformin targets

The instrument strength analysis suggested that instruments for the MC1, AMPK, GCG/GLP1, MG3, FBP1 and ADCY1 targets were deemed strong (F-statistics >10; Supplementary Table S6a). The instrument for GCG had an F-statistic = 1.40, which was excluded in the target-specific MR analysis with a caveat for potential weak instrument bias.

### Effects of perturbation of metformin targets on cardiometabolic outcomes

**Fig. 2:**
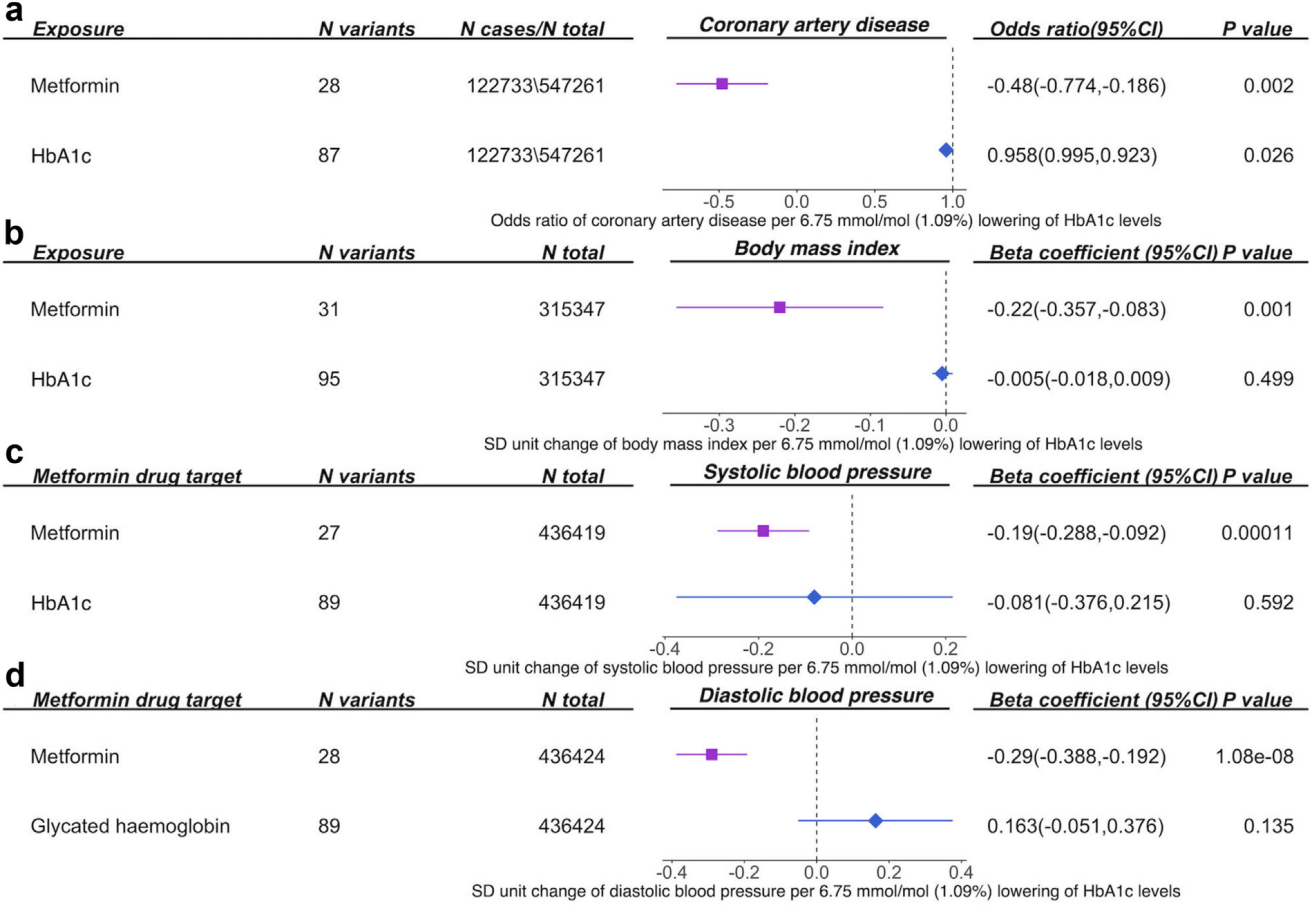
**Effects of perturbation of metformin targets and lowering of circulating HbA**_**1c**_**levels on(a)coronary artery disease,(b)body mass index,(c)systolic blood pressure and(d)diastolic blood pressure in the general population**. Both effect of perturbation of metformin and circulating HbA_1c_ effect were scaled to the same unit of 0.62% lowering of HbA_1c_ (which refers to 1 SD unit of HbA_1c_ levels) to allow direct comparison of the MR estimates.

Genetically proxied perturbation of metformin targets, equal to 1 standard deviation unit (SD, 0.62%) lowering of HbA_1c_, reduced CAD risk by 37.8% (OR = 0.62, 95% CI = 0.46–0.84, P = 0.002; Supplementary Table S8a), reduced BMI (β = −0.22, 95% CI = −0.35 to −0.09, P = 0.001), SBP (β = −0.19, 95% CI = −0.29 to −0.09, P = 1.07 × 10^−4^) and DBP levels (β = −0.29, 95% CI = −0.39 to −0.19, P = 1.08 × 10^−8^; Supplementary Table S8b, Fig. 2). Metformin targets showed little evidence of association with lipid phenotypes and atrial fibrillation (Supplementary Table S8). Our study also validated the overall effect of perturbation of metformin targets on reducing T2D risk in the general population (OR = 0.57, 95% CI = 0.35–0.92, P = 0.02; Supplementary Table S8a). The SBP and DBP data used in the main analysis adjusted for BMI in the original GWAS.^29^ A sensitivity analysis using SBP and DBP unadjusted for BMI still supports the potential role of metformin targets on these two blood pressure traits (Supplementary Table S8b). To minimize the influence of a target proxied by a single instrument, we conducted a sensitivity analysis excluding GDF15, GCG/GLP1 and MG3 instruments and only kept MC1 and AMPK instruments. This analysis still supports the effect of the two targets on reduced CAD risk and decreased BMI, SBP and DBP levels (Supplementary Table S8c).

In this study, we proxied the effect of metformin targets by using their HbA_1c_ lowering effect. This reflects a longer-term effect of blood glucose, but HbA_1c_ is also influenced by red blood cell traits. To control the impact of red blood cell traits on the MR estimates, we conducted a multivariable MR of metformin targets on cardiovascular disease (CVD) outcomes adjusted for red blood cell counts. The results still supported the putative causal roles of metformin targets on CAD, BMI, SBP and DBP (Supplementary Table S8d).

As a sensitivity analysis, we included two more targets of metformin, FBP1 and ADCY1, into the MR model (34 instruments in total) and estimated the effects of seven targets on the CVD outcomes. This analysis suggested that the effects of metformin targets was associated with reduced CAD and T2D risk, and decreased DBP, LDL-C and BMI levels (Supplementary Table S8e). These findings were similar with the general effect estimated using five metformin targets. The exception was LDL-C, in which LDL-C showed marginal evidence of an effect using five targets, but more robust evidence using seven targets.

As a benchmark, genetically proxied reduction in 1 SD unit of HbA_1c_ levels lowered CAD risk by 58.4% (OR = 0.42, 95% CI = 0.24–0.72, P = 0.001), lowered SBP levels (β = −0.58, 95% CI = −0.96 to −0.20, P = 0.003) and showed marginal evidence of lowering BMI levels (β = −0.28, 95% CI = −0.54 to −0.01, P = 0.04; Fig. 2). In contrast, there is little evidence to support the effect of lowering circulating HbA_1c_ on DBP (β = 0.09, 95% CI = −0.51 to 0.51, P = 0.93). Compared with effects of metformin targets, genetically proxied HbA_1c_ lowering tended to show larger effects on reducing CAD, BMI and SBP than metformin targets, although the confidence intervals do overlap (Fig. 2). In addition, genetically proxied lowered HbA_1c_ levels showed marginal effects on increased atrial fibrillation risk (OR = 1.43, 95% CI = 1.00–2.05, P = 0.05; Supplementary Table S9), but metformin targets showed little effect, which implies that these effects were unlikely to be influenced by the tested metformin targets.

### Target-specific effects of perturbation of metformin targets on cardiometabolic outcomes

Supplementary Fig. S3 presents the target-specific results of metformin targets on CAD, BMI, SBP and DBP. Most of the metformin targets showed protective effects on the four cardiometabolic outcomes with a few exceptions. For example, genetically proxied MC1-specific effect reduced CAD risk (OR = 0.65, 95% CI = 0.48–0.87, P = 0.004; Supplementary Fig. S4), BMI levels (β = −0.21, 95% CI = −0.34 to −0.08, P = 0.001; Supplementary Fig. S5) and DBP levels (β = −0.31, 95% CI = −0.42 to −0.20, P = 4.04 × 10^−8^), but increased SBP levels (β = 0.18, 95% CI = 0.07–0.29, P = 1.15 × 10^−3^), which suggested the possibility of conflicting effects of the same metformin target on different outcomes. The genetically proxied MG3-specific effect reduced SBP levels (β = −0.49, 95% CI = −0.84 to −0.14, P = 0.007) and marginally reduced DBP levels (β = −0.38, 95% CI = −0.76 to −0.01, P = 0.04). The genetically proxied GDF15-specific effect showed some evidence of reducing CAD risk, but with potential to suffer from weak instrument bias. The ADCY1-specific analysis provided evidence to support its effect on reducing BMI levels and decreased T2D risk, whilst FBP1-specific analysis showed evidence of an effect on LDL-C. The target-specific analyses provided little evidence of target-specific effects on lipid phenotypes and atrial fibrillation (Supplementary Table S10).

For our top findings, the five MR sensitivity analyses showed little evidence to support violation of the exclusion restriction MR assumption (Supplementary Tables S8–S10).

### One-sample MR and triangulation analyses of top findings

Table 1 presented the one-sample MR results of perturbation of metformin targets on BMI, SBP and DBP in non-diabetic participants in UK Biobank. The genetically-predicted metformin targets showed strong effects on HbA_1c_ levels (except GCG), which validated the reliability and power of the instruments. The one-sample MR analysis suggested that the weighted average HbA_1c_ lowering effect of seven metformin targets causes lower levels of BMI (β = −0.17, 95% CI = −0.28 to −0.07), SBP (β = −0.61, 95% CI = −1.07 to −0.14) and DBP (β = −0.90, 95% CI = −1.17 to −0.63) in non-diabetic individuals (Table 1). The target-specific one-sample MR analysis further indicated that the genetically-predicted HbA_1c_ lowering effects of MC1, AMPK and ADCY1 cause lower levels of BMI; HbA_1c_ lowering effects of MC1 and AMPK cause lower levels of SBP; while HbA_1c_ lowering effects of MC1 cause lower levels of DBP (Table 1). This target-specific analysis highlights the importance of MC1 and AMPK pathway on cardiometabolic health. The two-sample MR results using data from single cohort suggested consistent causal relationships between metformin targets and BMI, SBP and DBP. One additional finding was the HbA_1c_ lowering effect of FBP1 on DBP (β = −3.69, 95% CI = −6.21 to −1.22; Table 1).

**Table 1:** Effect of target specific HbA1c lowering effects of metformin targets on body mass index, systolic blood pressure and diastolic blood pressure in UK Biobank participants without type 2 diabetes.

Outcome	Targets	N of instruments	MR methods	Beta (95% CI)
Body mass index (kg/m^2^)	All metformin targets	34 SNPs	Inverse variance weighted (IVW)	−0.26 (−0.43, −0.09)
			IVW (fixed effects)	−0.26 (−0.34, −0.18)
			Weighted median	−0.09 (−0.22, 0.04)
			Weighted mode	−0.06 (−0.18, 0.05)
			Two stage least squares	−0.17 (−0.28, −0.07)
	AMPK	3 SNPs	Inverse variance weighted	−0.89 (−1.35, −0.44)
			IVW (fixed effects)	−0.89 (−1.35, −0.44)
			Two stage least squares	−1.14 (−2.02, −0.25)
	Mitochondrial complex I	27 SNPs	Inverse variance weighted	−0.21 (−0.39, −0.03)
			IVW (fixed effects)	−0.21 (−0.29, −0.12)
			Weighted median	−0.08 (−0.21, 0.06)
			Weighted mode	−0.06 (−0.19, 0.06)
			Two stage least squares	−0.14 (−0.25, −0.03)
	GDF15	1 SNP	Wald ratio	−0.47 (−1.24, 0.31)
			Two stage least squares	−0.57 (−1.42, 0.29)
	Fructose bisphosphatase 1	1 SNP	Wald ratio	−1.78 (−2.76, −0.8)
			Two stage least squares	−1.94 (−3.77, −0.11)
	Adenylyl cyclase	1 SNP	Wald ratio	−0.81 (−1.30, −0.33)
			Two stage least squares	−0.85 (−1.43, −0.27)
Systolic blood pressure (mmHg)	All metformin targets	34 SNPs	Inverse variance weighted	−0.85 (−1.36, −0.33)
			IVW (fixed effects)	−0.85 (−1.20, −0.49)
			Weighted median	−0.59 (−1.15, −0.04)
			Weighted mode	−0.47 (−0.99, 0.06)
			Two stage least squares	−0.61 (−1.07, −0.14)
	AMPK	3 SNPs	Inverse variance weighted	−3.24 (−5.30, −1.28)
			IVW (fixed effects)	−3.24 (−5.30, −1.28)
			Two stage least squares	−4.19 (−8.24, −0.13)
	Mitochondrial complex I	27 SNPs	Inverse variance weighted	−0.77 (−1.33, −0.2)
			IVW (fixed effects)	−0.77 (−1.14, −0.4)
			Weighted median	−0.6 (−1.18, −0.02)
			Weighted mode	−0.52 (−1.04, 0.01)
			Two stage least squares	−0.55 (−1.02, −0.07)
	GDF15	1 SNP	Wald ratio	0.19 (−3.19, 3.58)
			Two stage least squares	0.07 (−3.71, 3.82)
	Fructose bisphosphatase 1	1 SNP	Wald ratio	−2.54 (−6.91, 1.73)
			Two stage least squares	−2.23 (−7.07, 2.62)
	Adenylyl cyclase	1 SNP	Wald ratio	−0.21 (−2.34, 1.91)
			Two stage least squares	−0.18 (−2.45, 2.09)
Diastolic blood pressure (mmHg)	All metformin targets	34 SNPs	Inverse variance weighted	−0.91 (−1.21, −0.62)
			IVW (fixed effects)	−0.91 (−1.12, −0.71)
			Weighted median	−0.82 (−1.15, −0.5)
			Weighted mode	−0.71 (−0.99, −0.43)
			Two stage least squares	−0.90 (−1.17, −0.63)
	AMPK	3 SNPs	Inverse variance weighted	−1.52 (−3.02, −0.04)
			IVW (fixed effects)	−1.52 (−2.65, −0.4)
			Two stage least squares	−1.68 (−3.72, 0.36)
	Mitochondrial complex I	27 SNPs	Inverse variance weighted	−0.89 (−1.2, −0.59)
			IVW (fixed effects)	−0.89 (−1.11, −0.68)
			Weighted median	−0.82 (−1.15, −0.5)
			Weighted mode	−0.70 (−0.99, −0.41)
			Two stage least squares	−0.87 (−1.15, −0.59)
	GDF15	1 SNP	Wald ratio	1.25 (−0.69, 3.19)
			Two stage least squares	1.36 (−1.06, 3.77)
	Fructose bisphosphatase 1	1 SNP	Wald ratio	−3.69 (−6.21, −1.22)
			Two stage least squares	−3.50 (−7.38, 0.38)
	Adenylyl cyclase	1 SNP	Wald ratio	−0.81 (−2.03, 0.41)
			Two stage least squares	−0.98 (−2.33, 0.37)

Participants' age and sex, genetic array and top 10 PCs were adjusted.

**Fig. 3:**
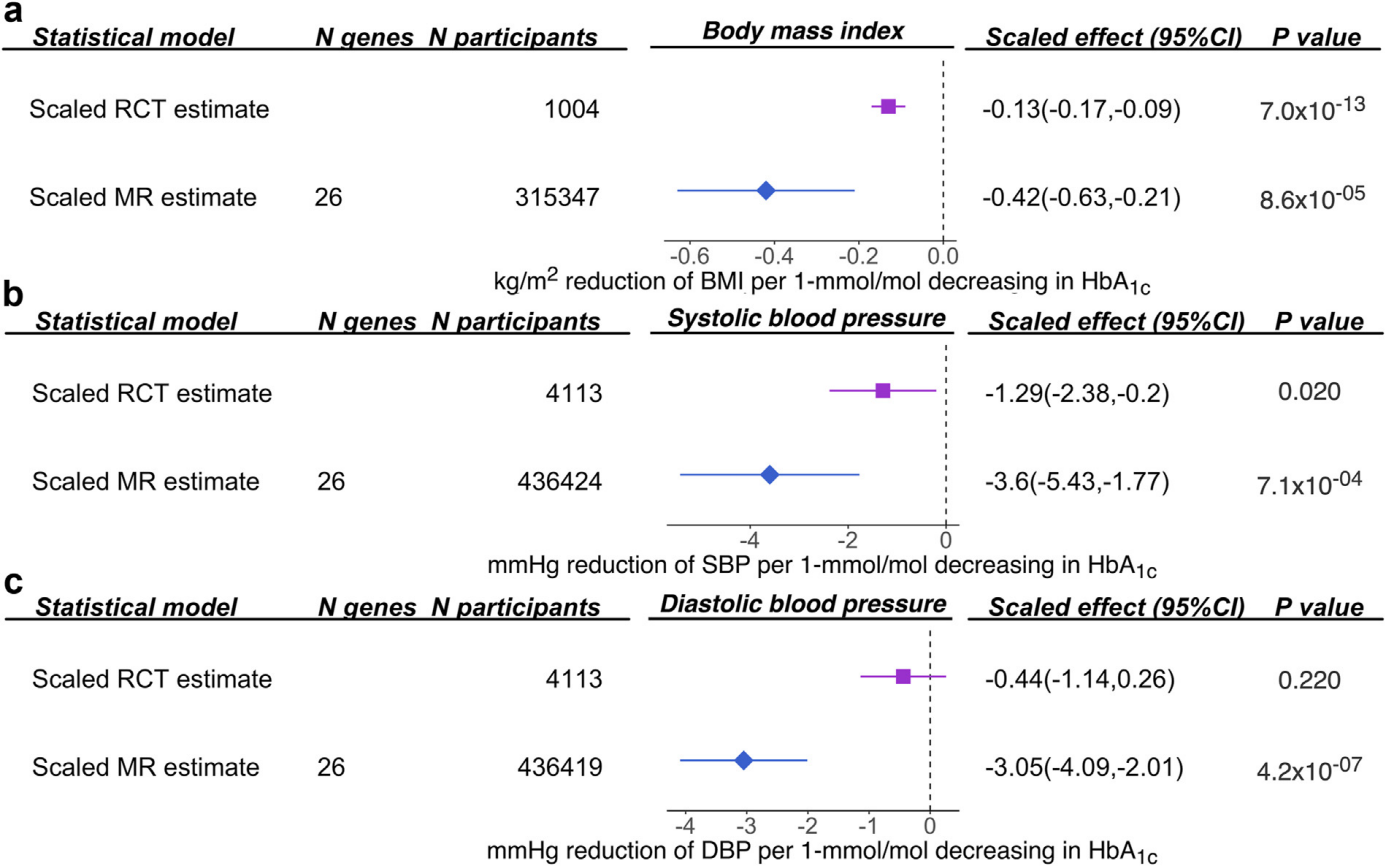
**Triangulation of clinical trial/observational and genetic evidence for perturbation of metformin targets on body mass index and diastolic blood pressure**. (a) The effect of perturbation of metformin targets on body mass index; (b) the effect of perturbation of metformin targets on systolic blood pressure; (c) the effect of perturbation of metformin targets on diastolic blood pressure. The clinical trial (RCT) or observational (observed) effect size and genetic (MR) effect size were re-scaled to the same unit, so these pieces of evidence are comparable.

We further triangulated the existing clinical trial evidence from the literature with the genetic evidence we obtained from the MR analysis (Supplementary Table S11). For BMI, SBP and DBP, evidence from the meta-analysis of trials and our MR analyses both demonstrated metformin effects consistent with a reduction in BMI and DBP (Fig. 3).

## Discussion

In this study, we estimated the effects of seven known metformin targets on eight cardiometabolic outcomes and have shown that the HbA_1c_ lowering effect of metformin targets leads to an improvement in a wide range of cardiometabolic conditions, including CAD, BMI and blood pressure, in the general population. To date, meta-analyses of clinical trials have suggested a beneficial effect of metformin on reducing cardiometabolic disease risk, but the decision to approve metformin for treating these conditions is still under investigation by the U.S. Food and Drug Administration (FDA). The one-sample MR further showed the effect of metformin targets on reducing BMI, SBP and DBP levels in non-diabetic individuals. This study therefore provides evidence to support the prioritization of metformin as a drug to improve cardiometabolic health in the general and non-diabetic population.

Previous epidemiological and MR studies have suggested that metformin and its target AMPK may protect against cardiovascular disease.^6^^,^^11^ The UK Prospective Diabetes trial (UKPDS) demonstrated that glucose lowering by metformin compared with only diet control reduced all-cause mortality and myocardial infarction in newly diagnosed T2D overweight patients.^37^ However, an existing meta-analysis of clinical trials suggested weak evidence to support the role of metformin on reducing risk of cardiovascular disease among individuals with diabetes.^38^ A recent MR study suggested the role of metformin activator, AMPK, on CAD.^11^ Our study provides two additional pieces of evidence: (1) the average effect of seven metformin targets on CAD in the general population; (2) the target specific effect of mitochondrial complex I on reducing CAD risk.

Existing epidemiological and preclinical studies have suggested that metformin has favourable effects in terms of reducing body weight.^39^ However, existing large-scale trials showed modest and inconsistent effects of metformin on weight loss.^40^ Due to this existing evidence, the US FDA has not approved metformin as a stand-alone weight loss agent. The current clinical guidelines do not recommend metformin as a monotherapy for obese patients without diabetes. However, off-label usage of metformin as an anti-obesity agent for non-diabetic individuals is relatively common in clinical practice. Our results using genetics suggested a weighted averaged causal effect of perturbation of metformin targets on reducing BMI levels, in the general and non-diabetic populations. Whether metformin could be suggested as a weight-loss agent to the general population, especially to those non-diabetic individuals at high risk of getting diabetes, is worthy of clinical investigation in future metformin trials.

For blood pressure, a few clinical trials among diabetic patients have shown little evidence of an effect of metformin on BP levels.^4^^,^^41^ A more recent meta-analysis of 28 trials consisting of 4113 non-diabetic participants suggested that metformin could effectively lower SBP in non-diabetic patients.^42^^,^^43^ In vivo studies have suggested a few potential mechanisms for metformin influencing blood pressure, which include some non-glycemic mechanisms such as reduction of intracytoplasmic calcium and improvement of endothelial function.^44^ Our study suggested that perturbation of metformin targets may have a general causal effect on reducing SBP and DBP levels in the general and non-diabetic populations. Future trials are needed to investigate the effect of metformin on blood pressure in non-diabetic individuals with dysglycemia and/or pre-diabetes symptoms.

The effects of HbA_1c_ on CVD outcomes were discussed in this study. For CAD, previous MR evidence and our results support a causal role of HbA_1c_ on CAD.^45^ For blood pressure, some studies support a causal role of blood pressure on diabetes,^46^^,^^47^ whilst our study suggests a causal role of HbA_1c_ on SBP. For body weight, BMI is a clear causal risk factor for a set of diseases including T2D.^48^ However, the effect of blood glucose on body weight is less clear and potentially non-causal. Collectively, the causal effect of blood glucose on blood pressure and body weight needs further investigation.

Our study has several strengths. First, our study validated the beneficial effects of several known metformin targets on heart disease, body weight and blood pressure. These findings may open opportunities to further investigate the target-specific effects of metformin. Second, our study aimed to understand the role of metformin targets on cardiometabolic health in non-diabetic individuals. It is known that the efficacy of metformin on HbA_1c_ reduction is not equal in diabetic and non-diabetic patients (1.14% vs 0.13%), with glucose-stimulated insulin secretion of metformin proposed as one of the underlying mechanisms.^49^ Our MR results suggest that metformin's efficacy on cardiometabolic conditions, especially on blood pressure and body weight, is likely to be true, even in those without diabetes. This may provide timely evidence to support some ongoing trials such as the VA-IMPACT trial, which aims to evaluate cardiovascular outcomes in patients with pre-diabetes and established CVD treated with metformin versus placebo (NCT02915198).

### Caveats and limitations

Our study also has limitations. First, by definition, MR estimates the effects of perturbation of metformin targets on the outcomes rather than the direct effect of metformin use. In this study, we systematically estimated the causal roles of known metformin targets on eight cardiometabolic outcomes. However, combining effects from different targets into a single MR does not necessarily reflect the true biological action of the drug because not all targets are likely to have been captured here, and the unmeasured targets could potentially change the results. In addition, our meta-analysis model assumes no strong interaction between the five metformin targets, which may not always be true. As a validation analysis, a factorial MR of AMPK and MC1 targets in the UK Biobank was conducted but underpowered (results not shown). Therefore, we did not systematically apply the interaction model in this study. Second, our MR estimates of the effect of perturbation of metformin targets were weighted by the precision of each target, and then scaled to represent reductions in HbA_1c_ levels rather than the direct effect of the drug target. This assumes that the effect of perturbation of metformin targets is proportional to HbA_1c_ lowering. Third, this study used HbA_1c_ data as exposure, which brings good statistical power. But the downside is that HbA_1c_ is also influenced by blood cell phenotypes (e.g. red blood cell counts). Our multivariable MR confirmed that the effects of metformin targets on cardiometabolic outcomes were still robust even after controlling for the influence of blood cell phenotypes. Fourth, although the MR and HbA_1c_ MR results implied the possibility of glycemic dependent and independent effect of metformin on cardiometabolic outcomes, we were not able to formally investigate this using multivariable MR since the metformin target effects and HbA_1c_ were both proxied by circulating HbA_1c_ levels from the same UK Biobank study. The non-glycemic causal mechanisms of metformin on cardiometabolic health are therefore worthy of further investigation. Fifth, GDF15 is a target of interest to endocrinologists. A recent study showed that the effect of metformin on body weight control was mediated by circulating levels of GDF15.^50^ Further studies suggested that GDF15 may also have beneficial effects on NAFLD and insulin resistance,^51^ due to the function of GDF15 in restraining energy intake.^52^, ^53^, ^54^, ^55^ In our study, the strength of GDF15 instruments was insufficient to provide robust evidence of a causal effect, although we still observed some evidence of a target-specific effect of GDF15 on CAD. Fifth, the one-sample MR was conducted in non-diabetic individuals, which represented as selection of samples and may increase the possibility of collider bias (also known as selection bias).^56^^,^^57^ A collider (e.g. diagnosis of diabetes) is a shared causal consequence of the exposure (e.g. HbA_1c_, a marker of diagnosis of diabetes) and outcome (e.g. BMI, as thinner people are less likely to be diagnosed with diabetes), which may induce spurious associations between the metformin targets and cardiovascular outcomes. However, the two-sample MR in the general population yields similar MR results, and is less likely to be influenced by this collider bias issue, suggesting our results were likely to be valid. Finally, the one sample MR estimate using two-stage least squares method is more prone to data overfitting than two-sample MR. In this study, we were inspired by a recent study that used two-sample MR methods in a single cohort.^33^ The results of the one-sample and two-sample MR estimates in non-diabetic individuals in the UK Biobank were generally similar. This implies that although the one-sample MR results were likely to be overfitted, the causal relationships between metformin targets and cardiometabolic phenotypes in non-diabetic individuals are likely to be true.

From a clinical perspective, our study suggests that these metformin targets are likely to have a causal role in reducing CVD burden in the general population and in non-diabetic individuals. This finding provides evidence to consider metformin as a potential intervention target for CVD prevention in non-diabetic individuals. This evidence supports further investigation of the efficacy of metformin targets with glucose levels, especially in those with pre-diabetes symptoms.

### Conclusions

Our results represent a comprehensive assessment of genetically proxied effects of perturbation of metformin targets on eight cardiometabolic outcomes. Our results provide evidence to support the general and target-specific effects of metformin on benefiting cardiometabolic health. The different effects of metformin use on cardiometabolic outcomes in different subgroups of participants need to be evaluated in future clinical trials.

## Contributors

J.Z., M.X., W.W., G.N., T.R.G. and Y.B. designed the study, wrote the research plan, and interpreted the results. J.Z. and Q.Y. have accessed and verified the underlying data. J.Z. undertook the main MR and sensitivity MR analyses with feedback from M.X., Q.Y. and C.Y.H. Q.Y. and C.Y.H. conducted the follow-up MR and wrote relevant sections. S.B., S.L.A.Y and S.L. supported the instrument selection. M.V.H supported the triangulation analysis. J.Z. wrote the first draft of the manuscript with critical comments and revision from M.X, Q.Y., V.W., J.L.L., J.Q.W., R.X.L., Y.X., T.W., Z.Y.Z., C.Y.H., J.Y., S.B., S.L.A.Y., S.L., E.A., M.V.H., G.D.S., G.N., W.W., T.R.G. and Y.B. J.Z. is the guarantor. The corresponding author attests that all listed authors meet authorship criteria and that no others meeting the criteria have been omitted. All authors read and approved the final version of the manuscript.

## Data sharing statement

The GWAS summary data used in this study are publicly available via the IEU OpenGWAS database (https://gwas.mrcieu.ac.uk/).

## Declaration of interests

T.R.G, J.Z. and G.D.S have received research funding from various pharmaceutical companies to support the application of Mendelian randomization to drug target prioritization. G.D.S. reports Scientific Advisory Board Membership for Relation Therapeutics and Insitro. M.V.H. is presently employed by 23andMe and holds stock in the company, and has previously consulted for Boehringer Ingelheim. The remaining authors have nothing to disclose.
