# Supplementary material for: Efficacy of metformin targets on cardiometabolic health in the general population and non-diabetic individuals: a Mendelian randomization study
Source: eBioMedicine. 2023 Sep 19;96:104803. doi: 10.1016/j.ebiom.2023.104803 (PMC10514430; doi:10.1016/j.ebiom.2023.104803)
Supplement: Supplementary Figures [file mmc2.docx]

**Supplementary Figures**

**
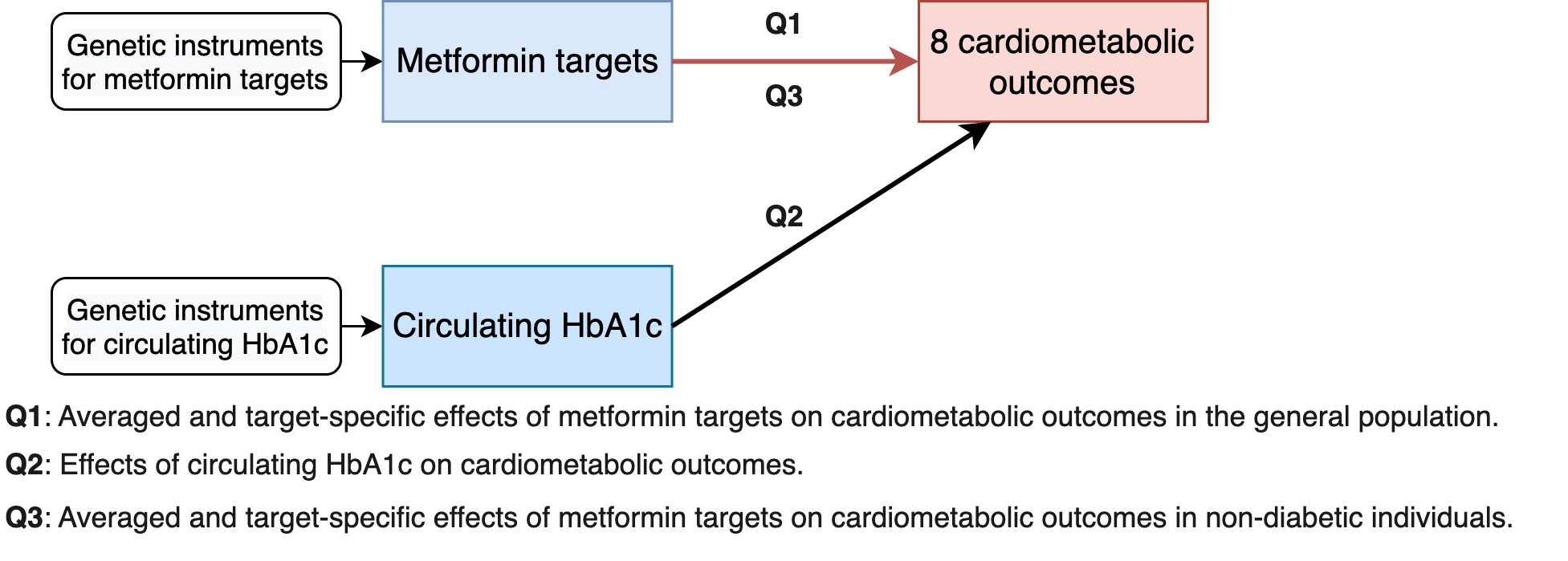
**

**Supplementary Fig. 1. The causal diagram between metformin, HbA1c and cardiometabolic phenotypes in the general population and non-diabetic individuals.**

**
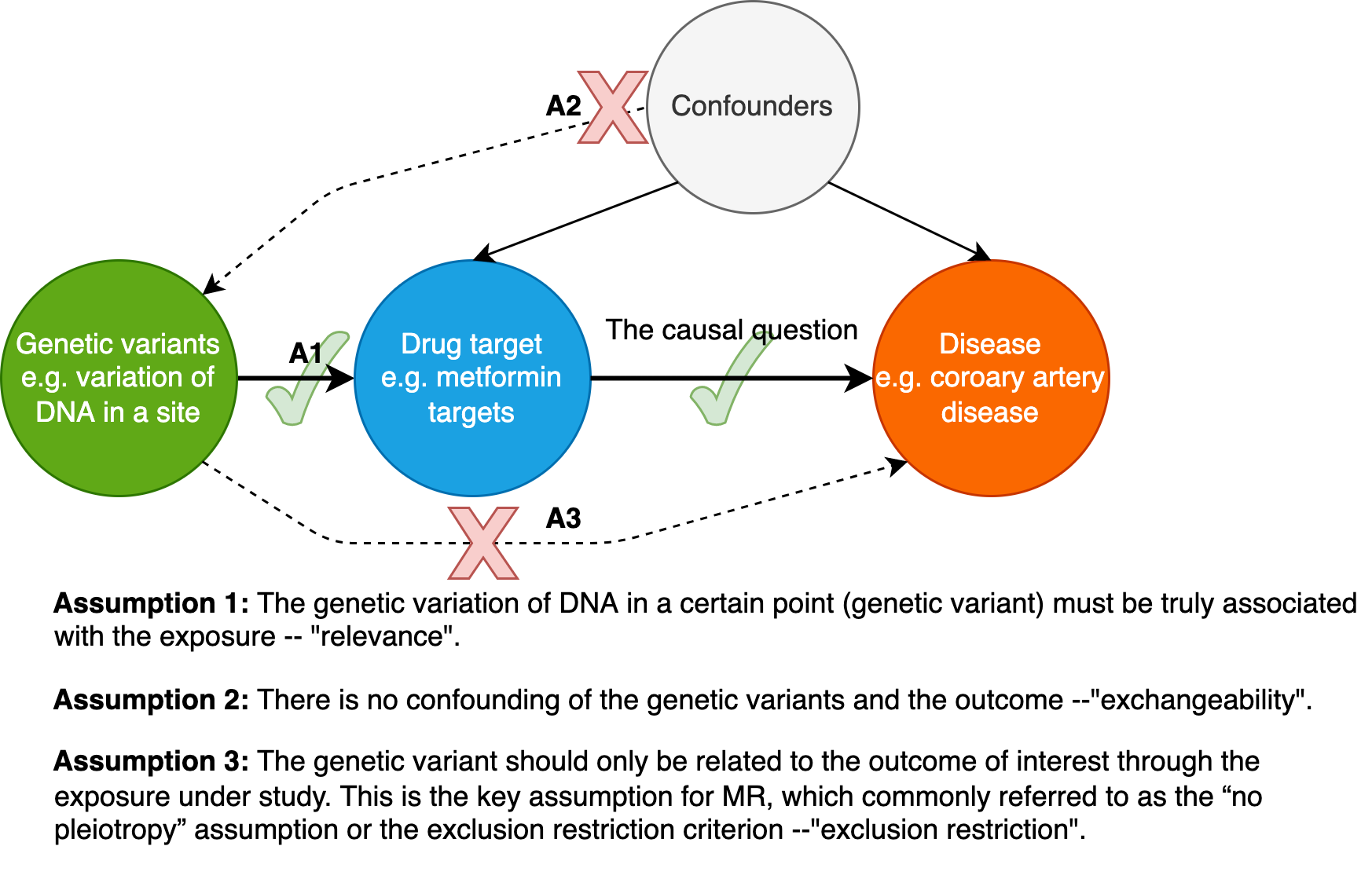
**

**Supplementary Fig. 2. Three core assumptions of Mendelian randomization.** Notation: horizontal pleiotropy, where a genetic variant is associated with multiple traits independently of the exposure of interest, potentially violates this assumption.

**
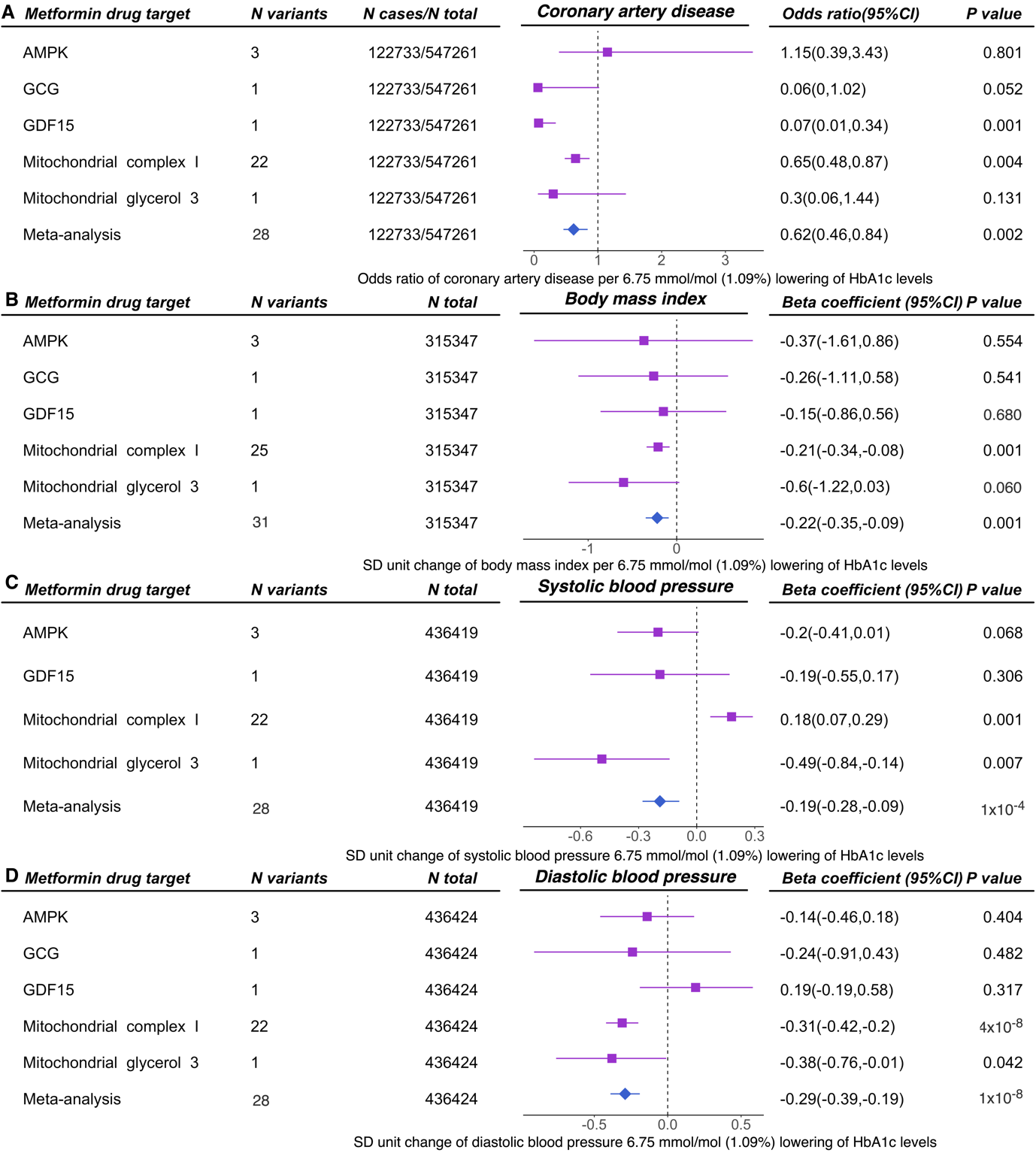
**

**Supplementary Fig. 3. General and target-specific effects of metformin targets on coronary artery disease, body mass index, systolic blood pressure and diastolic blood pressure in the general population.**

**
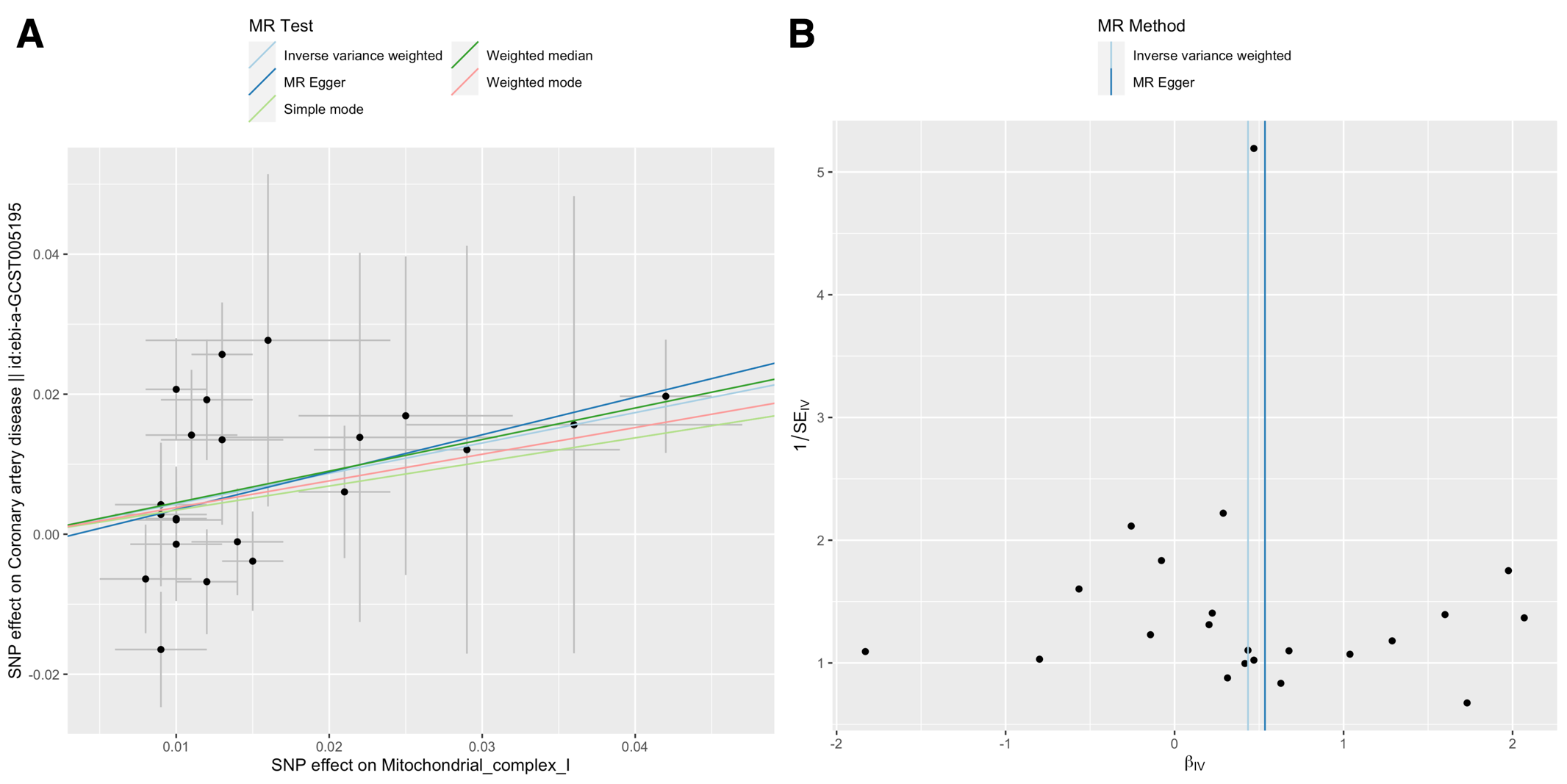
**

**Supplementary Fig. 4. Scatter and forest plots for the Mendelian randomization estimate of mitochondrial complex 1 on coronary artery disease.** (A) scatter plot for the effect of HbA1c lowering via mitochondrial complex 1 on coronary artery disease; (B) funnel plot of single SNP Mendelian randomization estimates.

**
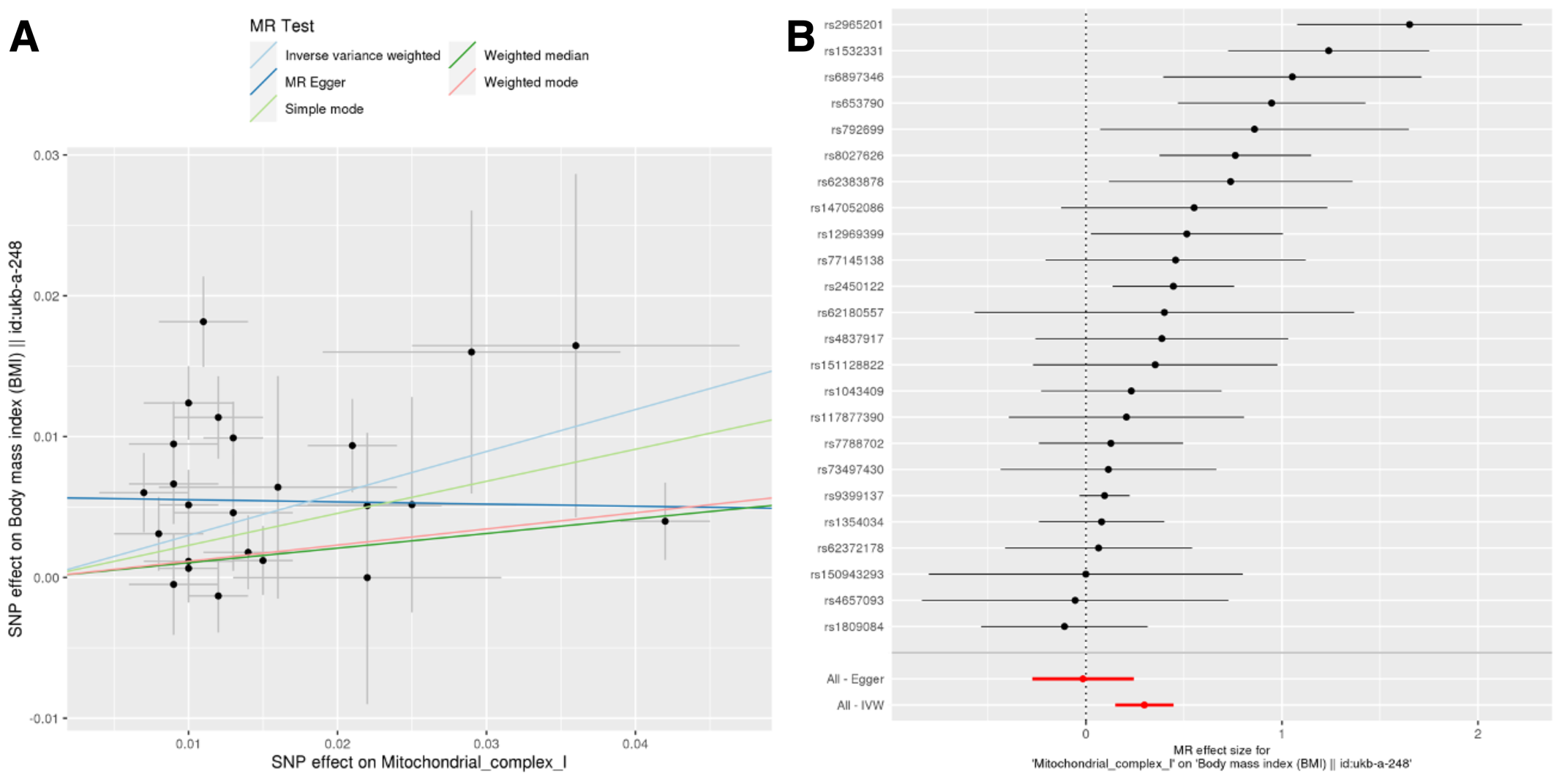
**

**Supplementary Fig. 5. Scatter and forest plots for the Mendelian randomization estimate of mitochondrial complex 1 on body mass index.** (A) scatter plot for the effect of HbA1c lowering via mitochondrial complex 1 on body mass index; (B) forest plot of single SNP Mendelian randomization estimates.

**Supplementary Note 1. Selection and validation of genetic instruments**

To select valid instruments for the five metformin targets, we first mapped the five targets to related genes using information from drug-gene interaction (1) and DrugBank (2). Using this approach, we identified 68 genes that related to metformin actions (58 genes for MC1; 1 gene for MG3; 7 genes for AMPK; 1 gene for GDF15 and 1 gene for GCG/GLP1). For the 68 metformin related genes, we identified genetic variants associated with the expression levels of the corresponding genes using data from recent GWAS studies of gene expression levels in 49 human tissues and protein expression levels in plasma(3)^,^(4)^,^(5)^,^(6)^,^(7)^,^(8)^,^(9). A p-value cut-off of 0.01 was used to select genetic variants associated with the expression levels of the 68 metformin genes. This selection process mapped 68 metformin-related genes to 2,021 candidate genetic instruments, which including MC1: 1,631 variants proxying MC1 (**Supplementary** **Table 1**), 48 variants proxying MG3 (**Supplementary** **Table 2**), AMPK: 292 variants proxying AMPK (**Supplementary** **Table 3**), 43 variants proxying GDF15(**Supplementary** **Table 4**), 6 variants proxying GCG/GLP1 (**Supplementary** **Table 5A**), 48 variants proxying ADCY1 (**Supplementary** **Table 5B**) and 48 variants proxying FBP1 (**Supplementary** **Table 5C**).

We further applied MR and genetic colocalization methods(10)^,^(11) to validate genetic instruments of metformin targets been selected for the MR analysis. In more details, the variant-expression associations were used as exposure, the variant-glucose associations were used as outcome to estimate the expression-HbA1c association via the tested variant in this validation analysis. Genetic colocalization was applied to confirm the expression-glucose associations via the tested variants were not biased by nearby genetic variants that in LD with the tested variants. For genetic variants with evidence of an effect on glycemic traits, we selected those with the lowest P value that had LD r^2^ less than 0.001. CEU specific LD among variants were estimated from the 1000 Genomes Project (phase 3) implemented in the two-sample MR package(12).

**Supplementary Note 2. One-sample Mendelian randomization of metformin targets on BMI, SBP and DBP in non-diabetic individuals**

We further conducted one-sample MR in UK Biobank to explore the linear effects of metformin targets on BMI, SBP and DBP. For exposure, weighted GRS for metformin targets (proxied by HbA1c) were derived by combining the 34 metformin instruments (**Supplementary** **Table 6A**). The target-specific effect of each metformin targets was also tested. For outcomes, individual level data for BMI (UKBB ID: 21001), SBP (UKBB ID: 4080) and DBP (UKBB ID: 4079) were extracted from European participants in UK Biobank. To fit with the two-stage least squares model, outliers with HbA1c, BMI, SBP or DBP levels outside four standard deviation unit from the mean were excluded. We restricted analysis to 360,347 unrelated European samples to control for the influence of population structure and relatedness. In addition, type 2 diabetes patients were excluded based on the following ICD10 codes:

• E10 (insulin-dependent diabetes mellitus)

• E11 (non-insulin-dependent diabetes mellitus)

• E12 (malnutrition-related diabetes mellitus)

• E13 (other specified diabetes mellitus)

• E14 (unspecified diabetes mellitus)

• O24 (diabetes mellitus in pregnancy)

After selection, 338,425 non-diabetic European individuals were included in this analysis.

**Supplementary Note 3. Literature search and triangulation of MR and trial evidence.**

For the three top MR findings, we searched PubMed (from inception to March 1, 2021) for meta-analyses evaluating the effects of metformin on BMI, SBP and DBP. The search was perfumed using the following combined text and MeSH terms: ‘metformin’, ‘blood pressure’, and ‘body mass index’, with no restriction on language (see search strategy in the **Appendix box**). If more than one meta-analysis was identified, we only included the latest study, the one based on randomized controlled trials, or the one reported appropriate pooled estimated effect that could be compared with the MR results.

| **Search** | **Query** | **Results** |
| --- | --- | --- |
| #1 | Search: **metformin [Text Word]** | [24,012](https://pubmed.ncbi.nlm.nih.gov/?term=metformin+%5BText+Word%5D&sort=) |
| #2 | Search: **metformin [MeSH Terms]** | [14,200](https://pubmed.ncbi.nlm.nih.gov/?term=metformin+%5BMeSH+Terms%5D&sort=) |
| #3 | Search: **((blood pressure [Text Word]) OR (SBP[Title/Abstract])) OR (DBP[Title/Abstract])** | [464,376](https://pubmed.ncbi.nlm.nih.gov/?term=%28%28blood+pressure+%5BText+Word%5D%29+OR+%28SBP%5BTitle%2FAbstract%5D%29%29+OR+%28DBP%5BTitle%2FAbstract%5D%29&sort=) |
| #4 | Search: **blood pressure [MeSH Terms]** | [312,350](https://pubmed.ncbi.nlm.nih.gov/?term=blood+pressure+%5BMeSH+Terms%5D&sort=) |
| #5 | Search: **(body mass index [Title/Abstract]) OR (body mass index [MeSH Terms])** | [253,169](https://pubmed.ncbi.nlm.nih.gov/?term=%28body+mass+index%5BTitle%2FAbstract%5D%29+OR+%28body+mass+index%5BMeSH+Terms%5D%29&sort=) |
| #6 | Search: **(meta-analysis [Title/Abstract]) OR (meta-analysis [MeSH Terms])** | [182,535](https://pubmed.ncbi.nlm.nih.gov/?term=%28meta-analysis%5BTitle%2FAbstract%5D%29+OR+%28meta-analysis%5BMeSH+Terms%5D%29&sort=) |
| #7 | Search: **(systematic review [Title/Abstract]) OR (systematic review [MeSH Terms])** | [183,812](https://pubmed.ncbi.nlm.nih.gov/?term=%28systematic+review%5BTitle%2FAbstract%5D%29+OR+%28systematic+review%5BMeSH+Terms%5D%29&sort=) |
| #8 | Search: **#1 OR #2** | [24,012](https://pubmed.ncbi.nlm.nih.gov/?term=%231+OR+%232&sort=) |
| #9 | Search: **#2 OR #3 OR #4 OR #5** | [885,992](https://pubmed.ncbi.nlm.nih.gov/?term=%233+OR+%234+OR+%236+OR+%237&sort=) |
| #10 | Search: **#6 OR #7** | [288,388](https://pubmed.ncbi.nlm.nih.gov/?term=%238+OR+%239&sort=) |
| #11 | Search: **#8 AND #9 AND #10** | [172](https://pubmed.ncbi.nlm.nih.gov/?term=%2310+AND+%2311+AND+%2312&sort=) |

**Appendix box**. Search strategy for the literature review.

The literature search identified 172 records, of which 40 non-meta-analyses were excluded after reviewing titles and abstracts. Three studies were induced after full-text screening. This included two meta-analyses of existing metformin treatment trials on BMI(15), SBP and DBP(16). The trial effect estimate of BMI were obtained from a meta-analysis of 21 trials in 1,004 obese individuals, while the effect estimate of SBP and DBP were obtained from a meta-analysis of 26 trials in 4,113 non-diabetic participants. The mean difference of metformin arm vs placebo arm was extracted from the papers. The genetic evidence was extracted from the two-sample MR of the overall metformin effects on BMI, SBP and DBP, where the genetic association information of the outcomes was obtained from large-scale GWASs of BMI, SBP and DBP (sample size equal to 336,107 and 317,756 separately).

We further rescaled the trial and MR estimates to a universal unit (reduction of BMI (kg/m^2^) or SBP/DBP (mmHg) per mmol/mol decreasing of HbA1c). To make the genetic evidence more comparable to the trial evidence, we processed the following steps that was used in previous studies(17)^,^(18):

1. For trial evidence:
   1. Obtained the causal effect of metformin use on HbA1c from a meta-analysis of 35 trials, which was equal to an average 12.32 mmol/mol reduction of HbA1c with average 1500 mg/day metformin treatment for 22 weeks(19).
   2. Extracted the mean difference of BMI, SBP and DBP reported in the two meta-analyses(15)^,^  with the actual unit of kg/m^2^ reduction of BMI by 1500 mg/day treatment for averagely 14 weeks and mmHg reduction of SBP and DBP by 1500 mg/day treatment of metformin for averagely 83 weeks
   3. Rescale the unit of mean difference to kg/m^2^ reduction of BMI (or mmHg reductio of DBP) per mmol/mol decreasing of HbA1c by metformin use, which the mean difference value was divided by the effect of metformin use on HbA1c (12.32 mmol/mol reduction of HbA1c).
2. For genetic evidence:
   1. Obtained the two-sample MR estimate with the units of SD unit change of BMI (or DBP) per SD unit decreasing of HbA1c via metformin pathway MC1
   2. Obtained the SD value of BMI (4.76 kg/m^2^), SBP (19.3 mmHg), DBP (10.5 mmHg) and HbA1c (6.75 mmol/mol) from the UK Biobank.
   3. Rescale the unit of the MR estimate to kg/m^2^ reduction of BMI (or mmHg reduction of SBP/DBP) per mmol/mol decreasing of HbA1c via metformin targets.

## References for Supplementary Notes

1. Cotto KC, Wagner AH, Feng Y-Y, et al. DGIdb 3.0: a redesign and expansion of the drug-gene interaction database. Nucleic Acids Res. 2018;46:D1068–D1073.

2. Wishart DS, Knox C, Guo AC, et al. DrugBank: a comprehensive resource for in silico drug discovery and exploration. Nucleic Acids Res. 2006;34:D668-72.

3. Sun BB, Maranville JC, Peters JE, et al. Genomic atlas of the human plasma proteome. Nature 2018;558:73–79.

4. Folkersen L, Fauman E, Sabater-Lleal M, et al. Mapping of 79 loci for 83 plasma protein biomarkers in cardiovascular disease. PLoS Genet. 2017;13:e1006706.

5. Suhre K, Arnold M, Bhagwat AM, et al. Connecting genetic risk to disease end points through the human blood plasma proteome. Nat. Commun. 2017;8:14357.

6. Yao C, Chen G, Song C, et al. Genome-wide mapping of plasma protein QTLs identifies putatively causal genes and pathways for cardiovascular disease. Nat. Commun. 2018;9:3268.

7. Emilsson V, Ilkov M, Lamb JR, et al. Co-regulatory networks of human serum proteins link genetics to disease. Science 2018.

8. Võsa U, Claringbould A, Westra H-J, et al. Unraveling the polygenic architecture of complex traits using blood eQTL meta analysis. BioRxiv 2018.

9. Aguet F, Barbeira AN, Bonazzola R, et al. The GTEx Consortium atlas of genetic regulatory effects across human tissues. BioRxiv, 2019.

10. Giambartolomei C, Vukcevic D, Schadt EE, et al. Bayesian test for colocalisation between pairs of genetic association studies using summary statistics. PLoS Genet. 2014;10:e1004383.

11. Zheng J, Haberland V, Baird D, et al. Phenome-wide Mendelian randomization mapping the influence of the plasma proteome on complex diseases. Nat. Genet. 2020;52:1122–1131.

12. Hemani G, Zheng J, Elsworth B, et al. The MR-Base platform supports systematic causal inference across the human phenome. Elife 2018;7.

13. Tian H, Mason AM, Liu C, Burgess S. Relaxing parametric assumptions for non-linear Mendelian randomization using a doubly-ranked stratification method. BioRxiv 2022.

14. Sun Y-Q, Burgess S, Staley JR, et al. Body mass index and all cause mortality in HUNT and UK Biobank studies: linear and non-linear mendelian randomisation analyses. BMJ 2019;364:l1042.

15. Pu R, Shi D, Gan T, et al. Effects of metformin in obesity treatment in different populations: a meta-analysis. Ther. Adv. Endocrinol. Metab. 2020;11:2042018820926000.

16. Zhou L, Liu H, Wen X, Peng Y, Tian Y, Zhao L. Effects of metformin on blood pressure in nondiabetic patients: a meta-analysis of randomized controlled trials. J. Hypertens. 2017;35:18–26.

17. Hemani G, Bowden J, Davey Smith G. Evaluating the potential role of pleiotropy in Mendelian randomization studies. Hum. Mol. Genet. 2018;27:R195–R208.

18. Schmidt AF, Swerdlow DI, Holmes MV, et al. PCSK9 genetic variants and risk of type 2 diabetes: a mendelian randomisation study. The Lancet Diabetes & Endocrinology 2017;5:97–105.

19. Hirst JA, Farmer AJ, Ali R, Roberts NW, Stevens RJ. Quantifying the effect of metformin treatment and dose on glycemic control. Diabetes Care 2012;35:446–454.
